# Opportunities for combining data of Estonian and Russian monitoring to reflect on water quality in large transboundary Lake Peipsi

**DOI:** 10.1016/j.jglr.2022.05.009

**Published:** 2022-08

**Authors:** Olga Tammeorg, Lea Tuvikene, Sergey Kondratyev, Sergey Golosov, Ilya Zverev, Olga Zadonskaya, Peeter Nõges

**Affiliations:** a Chair of Hydrobiology and Fisheries, Estonian University of Life Sciences, Kreutzwaldi 5, 51006 Tartu, Estonia; b Ecosystems and Environment Research Programme, Faculty of Biological and Environmental Sciences, University of Helsinki, Viikinkaari 1, 00014 Helsinki, Finland; c Institute of Limnology, Russian Academy of Sciences, ul. Sevast’yanova 9, St. Petersburg 199105, Russia; d State Hydrological Institute, 23, 2-ia liniia V.O., St. Petersburg 199053, Russia

**Keywords:** Lake Peipsi, Transboundary, Monitoring, Lake water quality assessment, Filling gaps

## Abstract

Lake Peipsi, one of the world’s largest lakes, is shared between Estonia and Russia. The water quality in different parts of the lake has so far been assessed independently. Here we explore opportunities for combining data of Estonian and Russian monitoring. For that, we 1) analysed the compatibility of data for some water quality variables; 2) estimated the potential effects of the differences in sampling frequency; 3) provided a few regression models to calculate the missing data for months not sampled by the Russian side. Data of the concurrent Estonian and Russian sampling indicated a good compatibility. Estonian data analysis suggested that water quality assessment results are sensitive to sampling frequency. For example, total phosphorus (TP) in the largest basin showed a long-term decreasing trend in three month data that disappeared when data for other months were added. Disregarding some months may lead to under- or overestimation of certain factors with no consistency in the response of different basins. Hence, data of the whole ice-free period are recommended for an adequate water quality assessment. Furthermore, we demonstrated that monthly values of the water quality variables of the same year are autocorrelated. Based on this, we filled the gaps in the long-term data and compiled a dataset for the whole lake that enables its most comprehensive use in water quality assessment and management. Long-term data revealed no water quality improvement of Lake Peipsi. Further reduction of the external nutrient load is needed. Eutrophication is sustained by high internal phosphorus load.

## Introduction

Human activities have increased the input of nutrients to waterbodies leading to accelerated eutrophication globally (Schindler et al., 2016, Spears et al., 2017). Lake water quality management requires accurate assessment of lake status and trends that is ensured by monitoring. In the case of transboundary waterbodies, water quality monitoring programmes provide a basis for international agreements regulating the use of these waters and for evaluation of compliance with such agreements (Bartram and Ballance, 1996). Lake Peipsi (Chudsko-Pskovskoye in Russian), a three-basin-complex located on the border between Estonia and Russia, is an example of such lakes.

Based on the agreement between the Republic of Estonia and the Russian Federation on the use and protection of the natural resources signed in 1997, the Joint Commission for the Protection and Sustainable Use of Estonian-Russian Transboundary Waters (JC) was established. JC is in charge of transboundary co-operation between the governments of the two countries, organising the exchange of environmental monitoring data and harmonisation of measurement methods between the two countries, broadening the opportunities for co-operation between scientific and public organisations on both sides and supporting public debate on transboundary waters.

One of the challenges in the management of the large transboundary Lake Peipsi is the lack of common status assessment criteria. This is one of the central issues that the monitoring group of the JC has been dealing with since the 2000s. Large efforts in methods comparison and harmonisation of monitoring data have been done. As a result, several parameters including the concentrations of total phosphorus (TP), dichromatic chemical oxygen demand (COD_Cr_), biological oxygen demand (BOD_5_) and Secchi depth (Secchi) were suggested as possible common metrics of the ecological status of Lake Peipsi. Both chlorophyll *a* (Chl *a*) and total nitrogen (TN) concentrations are acknowledged to be important, but there are not enough data for these variables yet (Russian monitoring programme included these variables since 2013 and 2016, correspondingly) to be combined. Moreover, water samples are collected on a monthly basis from May to October in Estonia and three times per ice-free season (in May, August and October) on the Russian side that hypothetically can affect water quality assessment.

The present study aimed at elucidating opportunities to combine data of Estonian and Russian routine environmental monitoring to reflect the water quality in the whole lake. First, we analysed if the data for TP, COD_Cr_, BOD_5_, Secchi, and phytoplankton biomass (PhytBM) collected by the counterparts are compatible. For that purpose, we used data of the joint expeditions (i.e. when Estonian and Russian experts sampled concurrently the same sampling sites). Based on the data of Estonian monitoring, we: 1) estimated the potential effects of the differences in sampling frequency on water quality assessment by analysing long-term trends for water quality variables; 2) provided a few simple regression models to calculate values of water quality variables for the three months of the ice-free period with the most limited data (i.e, June, July and September that are not sampled by the Russian side). Finally, we combined Estonian and Russian monitoring data and compared long-term trends in water quality variables available for the three common sampling months (May, August, October) with those for the six months (May-October). We focused here on the analysis of the ice-free season data. However, winter monitoring in large lakes has received much attention recently, particularly due to growing concern of climate change (Weyhenmeyer, 2001, Blank et al., 2009, Sharma et al., 2019, Ozersky et al., 2021). Hence, the current experience on the ice-free period may be extrapolated also to winter data in future, when more data will be available.

## Materials and methods

### Study site

**Fig. 1:**
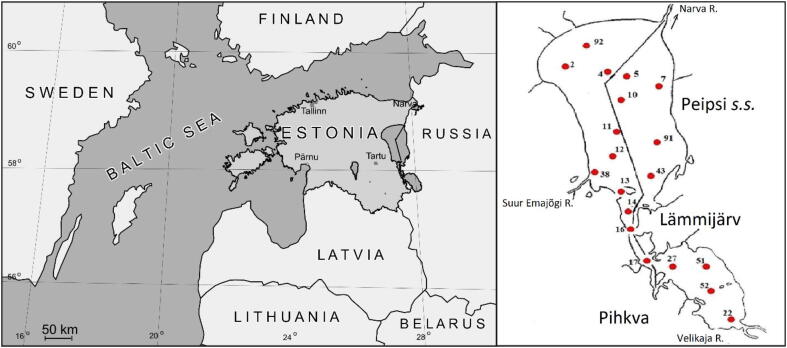
Location of Lake Peipsi and sampling sites.

By the surface area (3,555 km^2^), Lake Peipsi (Chudsko-Pskovskoye in Russian) belongs among the fifty largest lakes in the world (Herdendorf, 1982). Still it is a relatively shallow lake with a mean depth of 7.1 m and a maximum depth of 15.1 m. Ordinarily, Lake Peipsi is covered with ice from December to April. Though the lake is usually oxygen-rich during the ice-free period, anoxic conditions may occur in the bottom layers during the ice-covered period and on hot and calm summer days. The three basins of Lake Peipsi (Fig. 1) are different in terms of morphometry, hydrology, trophic state, and composition of biota (Kangur and Möls, 2007). The northern part, Lake Peipsi *sensu stricto* (*s.s.;* Chudskoye in Russian) is the largest (2,611 km^2^) and has the greatest mean depth (8.3 m). The southern parts of the lake system, i.e. Lake Lämmijärv (Teploye) and Lake Pihkva (Pskov), are considerably smaller and shallower than the northern part, Lake Lämmijärv 236 km^2^, mean depth 2.6 m, and Lake Pihkva 708 km^2^, mean depth 3.8 m. Both Estonian and Russian names are internationally accepted, though we used Estonian names hereafter in the text. Lake Lämmijärv is situated almost entirely in Estonia while Lake Pihkva is in Russia. The southern basins are more eutrophic than the northern basin.

The Rivers Velikaya and Emajõgi account for the bulk of the nutrient loading into the lake (Loigu et al., 2008). Pronounced decrease in external nutrient loading resulted from a marked reduction in the use of fertilisers in the 1990s. On the Estonian side, fertiliser application in 2001 was only 11% of that in the late 1980s and livestock production decreased about two-fold, also water consumption by people and industries lowered and wastewater treatment improved (Iital et al., 2005). Nõges et al. (2020) underlined a slight increase in the application of fertilisers together with the recovery and restructuring of agriculture in the independent Republic of Estonia, notably so from the early 2000s onwards when Estonia joined the EU and the agricultural subsidies enabled wider use of agrochemicals. Still, external TN loading from the whole catchment decreased by 19% and TP loading by 37% (Table 1; Zadoskaya et al., 2017). About 2/3 of the external TP loading and about half of the external TN loading is coming from the Russian side of the catchment (Zadoskaya et al., 2017).

**Table 1:** External loading of total nitrogen (TN) and total phosphorus (TP; tonnes per year) to Lake Peipsi (data presented at the annual meeting of the Joint Commission, summarized by Zadoskaya et al., 2017.

**Period**	Russian side				Estonian side				**Whole catchment**	
	TN	%	TP	%	TN	%	TP	**%**	**TN**	**TP**
2001–2005	9535	53	481	64	8325	47	270	36	17860	751
2006–2010	8143	45	442	63	9815	55	258	37	17958	700
2011–2015	7339	51	313	66	7180	49	160	34	14519	473

### Sampling

Under the Estonian monitoring programme, hydrochemical and hydrobiological water samples were collected predominantly from six stations in the pelagic zone of the Estonian side of Lake Peipsi (Fig. 1) monthly from May to October of 2001–2019. Chemical parameters were analysed by Estonian Environmental Researches Ltd. (Tartu, Estonia), and hydrobiological parameters by the Institute of Environmental and Agricultural Sciences of the Estonian University of Life Sciences. The Russian monitoring programme covers nine monitoring stations sampled in May, August and October. Since 2001, joint Estonian-Russian expeditions on the whole lake are carried out in August and March with as much as possible simultaneous sampling at all stations on both sides. In the current study, we used monitoring data for surface layer (0.5 m) TP, COD_Cr_, and BOD_5_. Water for determining integrated phytoplankton biomass (PhytBM) was taken with a 2-litre Limnos type sampler at 1-meter intervals from surface to 0.5 m above the bottom and mixed in a tank. Subsamples for phytoplankton identification were obtained directly from the tank, preserved with Lugol’s acidified iodine solution and counted under an inverted microscope using the Utermöhl (1958) technique. Biomass (wet weight) was estimated from the volumes of algal cells. The concentrations of TP, BOD_5_, and COD_Cr_ were determined following the standards ISO 15681–2, EVS-EN 1899–2, and SFS 3036, correspondingly. On the Russian side, hydrochemical samples were analysed by the Centre for Hydrometeorological Services of Pskov and hydrobiological samples by the North-West Administration on Hydrometeorology and Environmental Monitoring of the Russian Federation. Generally, the methods of sampling and determination are the same as on the Estonian side for the studied chemical variables. Less intercalibration was carried out for biological variables, and Chl *a* was the only one that showed consistency (comparable results). Here, we study also data compatibility for PhytBM (analysed similarly in shared countries) with considerably longer series than Chl *a*.

### Statistical methods and analyses

Pairwise *t*-test was used to analyse the differences in monitoring data for TP, COD_Cr_, BOD_5_, Secchi, and PhytBM obtained by Estonian and Russian experts during joint expeditions in August 2003–2014 (i.e., data sampled concurrently from the same sampling sites).

Long-term trends of TP, COD_Cr_, BOD_5_, Secchi, and PhytBM based on three measurements (May, August, October) and six measurements (May-October) were studied with linear models using Estonian monitoring data to discover the potential impact of sampling frequency on lake water quality assessment. For the Estonian side, we report also the trends for Chl *a* and TN, as there are long-term data available. For the variables that showed differences in trends on three and six-month-data, we analysed also trends in long-term data for each of the six months (May-October) to see contribution of the month to the trend over the whole ice-free period. For the potential role of water temperature and water level, the long-term trends in May-October data (measured on the shore of Lake Peipsi *s.s.*) were also analysed. Additionally, we studied the potential of the data for the commonly sampled months (May, August and October) to predict values for the months with the most restricted data (June, July, September) with linear regression models. The best regression model was used to calculate the monthly values for June, July and September for the Russian side suggesting a similar seasonality.

Estonian and Russian monitoring data were combined (1) for the common sampling months (May, August, October), and (2) for six months (May-October, Russian data partly from regression models) to analyse the long-term (2001–2019) trends for the whole lake. Trends were studied with linear models. For the variables that appeared to be sensitive to sampling frequency, trends were analysed also in the monthly (May-October) long-term data. The seasonal dynamics were shown with the box-plot diagrams. The differences between basins in water quality variables were studied with analysis of variance using the Tukey post-hoc test. Additionally, we studied Spearman’s correlations between the variables.

## Results

### Compatibility of the sharing countries’ monitoring data

**Fig. 2:**
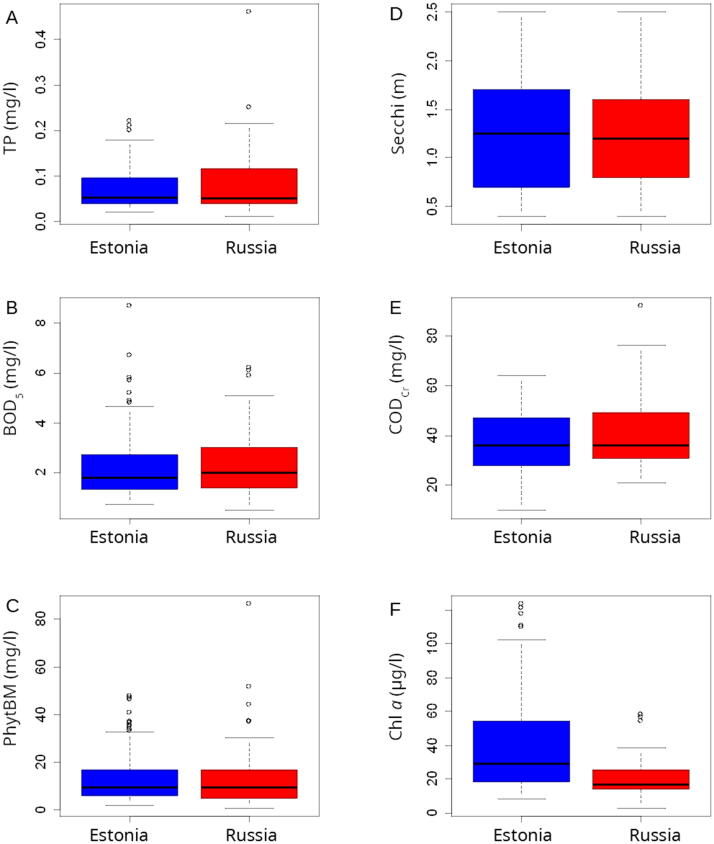
Distribution of the concurrent measurements from the same sampling sites by the Estonian and Russian experts during shared monitoring. The water quality variables included total phosphorus (TP; A), biological oxygen demand (BOD_5_; B), phytoplankton biomass (PhytBM; C), Secchi depth (Secchi; D), dichromatic chemical oxygen demand (COD_Cr_; E), and chlorophyll *a* (Chl *a*; F).

Data on studied variables collected by Estonian and Russian experts were reasonably similar (Fig. 2). There were no significant differences in data for PhytBM (Fig. 2C), Secchi (Fig. 2D) and BOD_5_ (Fig. 2B). Russian monitoring resulted in significantly higher values for TP (*p <* 0.01; Fig. 2A) and COD_Cr_ (*p <* 0.01; Fig. 2E). Nevertheless, these differences (0.010 mg/l and 2.77 mg/l, respectively) were much smaller than the standard deviations of the variables (Table 2, Table 3). Hence, Estonian and Russian monitoring data can be combined to obtain the full picture about the lake water quality in Lake Peipsi. Noteworthy, the present results indicated also a potential suitability of phytoplankton biomass to reflect the ecological state of the lake, as there are no long-term data for Chl *a* yet.

**Table 2:** Long-term (2001–2019) trends found in Estonian monitoring data of Lake Peipsi. Mean for the ice-free period is calculated using (1) six monthly measurements (May-October) and (2) three measurements from May, August, and October as in the Russian monitoring programme. Significance level is indicated with asterisk (*p <* 0.05*, *p <* 0.01^**^, *p <* 0.001^***^). The magnitude of trend indicates a change (decrease “-“ or increase “+”) of a variable in a unit per year. The water quality variables included total phosphorus (TP), chlorophyll *a* (Chl *a*), Secchi depth (Secchi), phytoplankton biomass (PhytBM), dichromatic chemical oxygen demand (COD_Cr_), biological oxygen demand (BOD_5_), and total nitrogen (TN).

		Six months			Three months		
Variable	Basin	Mean (SD)	Trend	*n*	Mean (SD)	Trend	*n*
TP (mg/l)	Peipsi *s.s.*	0.039(0.016)	n.s.	496	0.041(0.017)	-^**^	250
	Lämmijärv	0.075(0.026)	n.s.	214	0.075(0.029)	n.s.	108
Chl *a* (µg/l)	Peipsi *s.s.*	22.21(13.86)	n.s.	516	22.59(14.18)	n.s.	262
	Lämmijärv	37.65(20.54)	n.s.	226	39.10(21.04)	n.s.	114
Secchi (m)	Peipsi *s.s.*	1.77(0.54)	−0.010*	488	1.74(0.54)	n.s.	247
	Lämmijärv	0.91(0.24)	−0.014^***^	216	0.92(0.25)	−0.013^**^	110
PhytBM (mg/l)	Peipsi *s.s.*	6.3(4.07)	−0.101^**^	517	6.32(4.19)	−0.138^**^	264
	Lämmijärv	12.73(10.57)	n.s.	227	13.44(12.65)	n.s.	115
COD_Cr_ (mg/l)	Peipsi *s.s.*	30.46(8.14)	n.s.	492	31.37(8.64)	n.s.	250
	Lämmijärv	38.43(8.53)	n.s.	213	39.53(9.21)	n.s.	108
BOD_5_ (mg/l)	Peipsi *s.s.*	1.65(0.72)	+0.043^***^	512	1.64(0.68)	+0.039^***^	262
	Lämmijärv	2.35(1.07)	+0.027*	224	2.25(1.06)	n.s.	114
TN (mg/l)	Peipsi *s.s.*	0.697(0.208)	n.s.	496	0.707(0.216)	n.s.	250
	Lämmijärv	0.855(0.216)	n.s.	214	0.902(0.218)	−0.009*	108

**Table 3:** Regressions between water quality variables in June, July and September (that are missing in Russian monitoring) and those in May, August, and October (that are present also in Russian monitoring) based on data of Estonian monitoring. Logarithmic values were used. The regression models that were chosen to predict missing values of the Russian monitoring for June, July and September (using corresponding values for *Slope* and intercept, *Int*) are marked in bold. Significance level is indicated with asterisk (*p <* 0.05*, *p <* 0.01^**^, *p <* 0.001^***^). The water quality variables included total phosphorus (TP), Secchi depth (Secchi), phytoplankton biomass (PhytBM), dichromatic chemical oxygen demand (COD_Cr_), biological oxygen demand (BOD_5_), and chlorophyll *a* (Chl *a*).

Variable		Jun			Jul			Sep		
		*R^2^*	*Slope*	*Int*	*R^2^*	*Slope*	*Int*	*R^2^*	*Slope*	*Int*
TP (mg/l)	May	0.524^***^	**0.835**	**−0.481**	0.485^***^	0.905	−0.139	0.332^***^	0.576	−0.836
	Aug	0.524^***^	0.676	−1.401	0.534^***^	**0.762**	**−1.050**	0.533^***^	**0.533**	**−1.290**
	Oct	0.386^***^	0.726	−1.189	0.348^***^	0.758	−0.978	0.356^***^	0.568	−1.119
Secchi (m)	May	0.537^***^	**0.811**	**0.016**	0.551^***^	0.789	−0.079	0.343^***^	0.548	−0.081
	Aug	0.537^***^	0.796	0.313	0.601^***^	**0.816**	**0.195**	0.413^***^	**0.591**	**0.112**
	Oct	0.399^***^	0.763	0.240	0.459^***^	0.803	0.128	0.395^***^	0.626	0.028
PhytBM (mg/l)	May	0.165^***^	0.402	0.836	0.143^***^	0.312	1.540	0.003^n.s.^	0.047	2.080
	Aug	0.186^***^	**0.429**	**0.423**	0.224^***^	**0.394**	**1.084**	0.308^***^	0.513	0.969
	Oct	0.168^***^	0.519	0.453	0.076^**^	0.300	1.457	0.337^***^	**0.691**	**0.887**
COD_Cr_ (mg/l)	May	0.066^**^	0.251	2.497	0.006^n.s.^	0.087	3.160	0.043*	0.190	2.793
	Aug	0.196^***^	**0.376**	**2.028**	0.054^n.s.^	0.215	2.698	0.194^***^	**0.347**	**2.221**
	Oct	0.113^***^	0.269	2.436	0.041^n.s.^	0.182	2.834	0.184^***^	0.318	2.359
BOD_5_ (mg/l)	May	0.050*	0.248	0.393	0.198^***^	**0.584**	**0.361**	0.013^n.s.^	0.125	0.311
	Aug	0.054*	0.220	0.387	0.138^***^	0.418	0.380	0.060^**^	0.228	0.211
	Oct	0.201^***^	**0.502**	**0.395**	0.018^n.s.^	0.179	0.635	0.218^***^	**0.503**	**0.243**
Chl *a* (μg/l)	May	0.140^***^	0.401	1.631	0.147^***^	0.370	2.068	0.010^n.s.^	0.078	3.146
	Aug	0.102^***^	0.350	1.540	0.166^***^	**0.404**	**1.719**	0.090^***^	0.286	2.384
	Oct	0.158^***^	**0.463**	**1.281**	0.022^n.s.^	0.162	2.580	0.459^***^	**0.705**	**1.150**

### Effects of sampling frequency on the trends in long-term data based on Estonian data

Estonian monitoring data revealed that some information may be lost by omitting three months of the May-October period. Mean values over the years 2001–2019 were generally similar in both cases, i.e., averaging (1) over three months, and averaging over six months of the ice-free season (Table 2). However, long-term trends of Secchi, BOD_5_ and TP were affected by sampling frequency. First of all, averaging six-month data resulted in a significant decline in Secchi in Lake Peipsi *s.s.*, while no such trend was observed when data was averaged over three months. Secondly, BOD_5_ showed a long-term increase in Lake Lämmijärv when six measurements were considered that was not apparent in three month data. Interestingly, averaging three TP measurements resulted in a long-term declining trend in Lake Peipsi *s.s.*, but no trend was observed if data of six measurements per ice-free season were used.

**Fig. 3:**
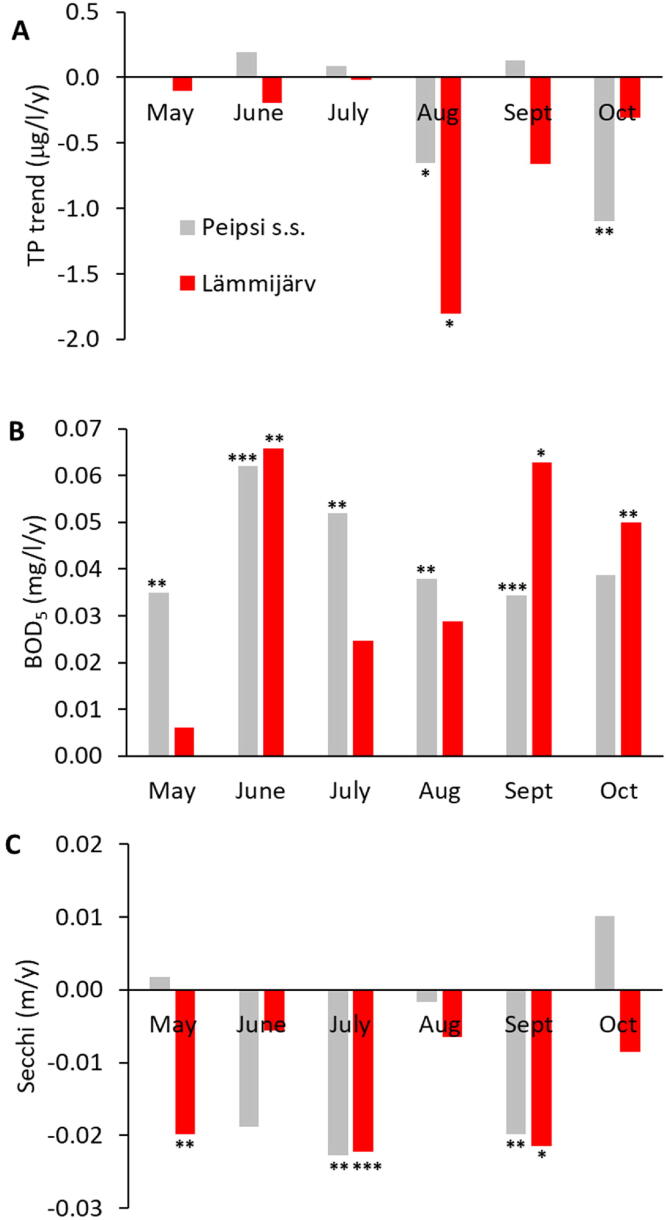
Changes in monthly trends of total phosphorus (TP; A), biological oxygen demand (BOD_5_; B) and Secchi depth (Secchi; C) in Lake Peipsi *s.s.* (grey), Lake Lämmijärv (red) in 2001–2019 based on Estonian monitoring data. Significance level is indicated with asterisk (*p <* 0.05*, *p <* 0.01^**^, *p <* 0.001^***^). (For interpretation of the references to colour in this figure legend, the reader is referred to the web version of this article.)

Over the years 2001–2019, TP showed a decline in August (Electronic Supplementary Material (ESM) Fig. S1D) and October (ESM Fig. S1F) in Lake Peipsi *s.s.* and in August (ESM Fig. S1D) in Lake Lämmijärv (Fig. 3A). BOD_5_ increased in all months from May to October (ESM Fig. S2A-F) in Lake Peipsi *s.s.*, while only in June (ESM Fig. S2B), September (ESM Fig. S2E) and October (ESM Fig. S2F) in Lake Lämmijärv (Fig. 3B). Secchi depth showed decreasing trends in July and September in Lake Peipsi *s.s.*, and in May, June and September in Lake Lämmijärv (Fig. 3C). Over the same period, water temperature showed an increasing trend in May, June, September and October and a decreasing trend in July and August (ESM Table S1). Water level in months May-September decreased, and there was no clear trend in October data (ESM Table S1).

Data for June, July and September (missing in the Russian monitoring) could be predicted from the data of May, August and October in most cases (Table 3). The strongest regressions were obtained for TP and Secchi. For both variables, May values were most suitable for estimating June values and August values for estimating July and September values. For BOD_5_ and Chl *a*, June and September values could be predicted best from October data, and July values from August data. COD_Cr_ was the only variable that could not be estimated for July based on May, August, October data, and correlated best with the June values (R^2^ = 0.151, p < 0.001, Slope = 0.431, Intercept *=* 3.160). As June is missing in Russian monitoring data, it should be predicted first from August values.

### Long-term changes in water quality of the whole Lake Peipsi based on the combined data (three-month and six-month-data)

The trends found in Estonian data for three months remained the same in Lake Lämmijärv when Russian data were added (compare Table 2, Table 4), because most sampling stations in this part of the lake are located on the Estonian side. Adding Russian data affected trends in Lake Peipsi *s.s.* for COD_Cr_ (from no trend to negative) and PhytBM (from negative to no trend; Table 4), suggesting larger spatial heterogeneity in these variables. COD_Cr_ is likely associated with wetlands that are sources of humic substances and are abundant on the Russian side of the Peipsi *s.s.* catchment.

**Table 4:** Trends found for the long-term (2001–2019) combined Russian and Estonian monitoring data for the whole Lake Peipsi. Mean for the ice-free period is based on 1) three measurements (May, August, October) to have comparable data for all three basins. Lake Pihkva is sampled only three months per vegetation period; 2) six values, i.e. three measurements and three predictions for the missing months (June, July and September). Significance level is indicated with asterisk (*p <* 0.05*, *p <* 0.01^**^, *p <* 0.001^***^). The magnitude of trend indicates a change (decrease “-“ or increase “+”) of a variable in a unit per year. The water quality variables included total phosphorus (TP), Secchi depth (Secchi), phytoplankton biomass (PhytBM), dichromatic chemical oxygen demand (COD_Cr_), biological oxygen demand (BOD_5_).

Variable	Basin	Mean (SD)	Trend	*n*	Mean (SD)	Trend	*n*
TP (mg/l)	Peipsi *s.s.*	0.040 (0.018)	−0.0004^**^	568	0.040 (0.017)	n.s.	1043
	Lämmijärv	0.071 (0.028)	n.s.	179	0.066 (0.027)	n.s.	317
	Pihkva	0.100 (0.063)	n.s.	222	0.090 (0.049)	n.s.	450
Secchi (m)	Peipsi *s.s.*	1.75 (0.51)	n.s.	551	1.75 (0.47)	n.s.	1013
	Lämmijärv	0.92 (0.24)	n.s.	178	0.96 (0.26)	n.s.	319
	Pihkva	0.87 (0.25)	+0.007^**^	214	0.91 (0.22)	+0.008^***^	430
PhytBM (mg/l)	Peipsi *s.s.*	6.28 (5.41)	n.s.	503	6.05 (5.11)	n.s.	930
	Lämmijärv	12.15 (11.04)	n.s.	166	11.05 (9.88)	n.s.	303
	Pihkva	11.04 (14.60)	n.s.	170	9.27 (11.33)	n.s.	344
COD_Cr_ (mg/l)	Peipsi *s.s.*	31.46 (9.40)	−0.314^***^	563	30.95 (8.23)	−0.228^***^	1034
	Lämmijärv	39.28 (9.29)	n.s.	177	37.52 (8.63)	n.s.	315
	Pihkva	45.21 (12.38)	n.s.	218	39.45 (10.54)	n.s.	446
BOD_5_ (mg/l)	Peipsi *s.s.*	1.76 (0.76)	+0.027^***^	580	1.77 (0.69)	+0.029^***^	1030
	Lämmijärv	2.33 (1.04)	n.s.	185	2.26 (0.96)	+0.026^**^	328
	Pihkva	2.49 (1.16)	+0.053^***^	222	2.26 (0.93)	+0.037^***^	442

**Fig. 4:**
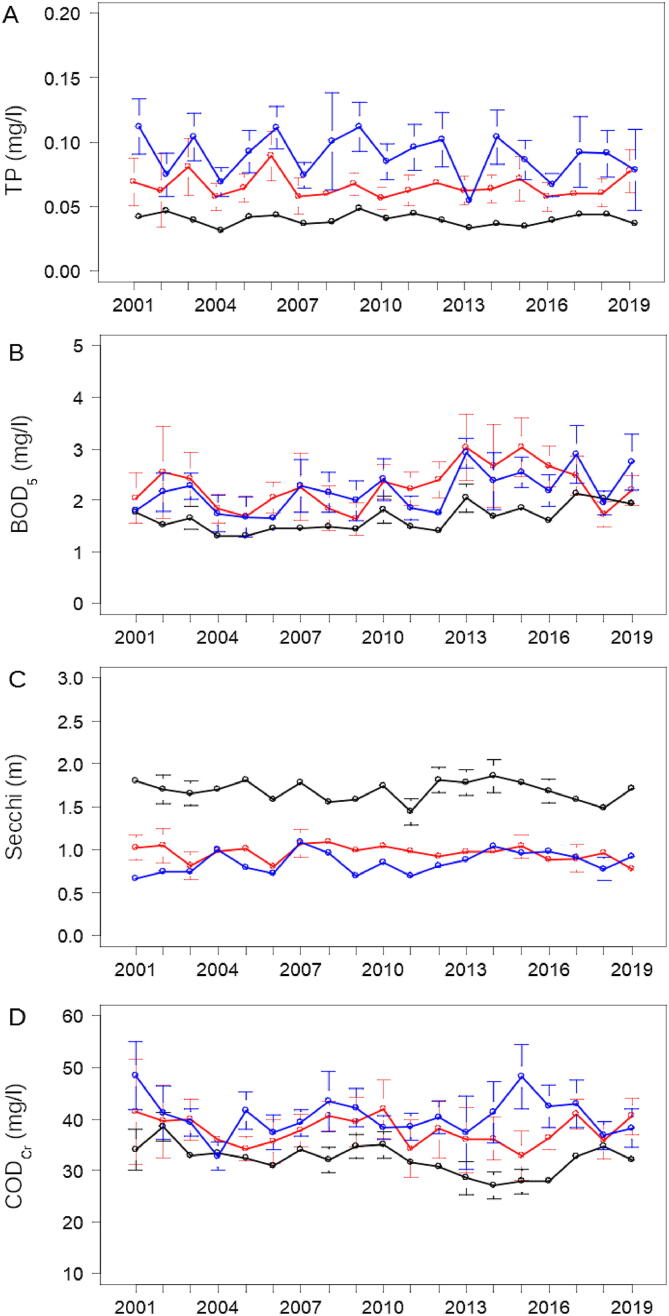
Long-term dynamics of total phosphorus (TP; A), biological oxygen demand (BOD_5_; B), Secchi depth (Secchi; C) and dichromatic oxygen demand; COD_Cr_; D) averaged over six months in Lake Peipsi *s.s.* (black), Lake Lämmijärv (red) and Lake Pihkva (blue) based on the combined data of Estonian and Russian monitoring. (For interpretation of the references to colour in this figure legend, the reader is referred to the web version of this article.)

The three-month averaged Estonian-Russian joint data showed a slight long-term decline in TP and COD_Cr_, and an increase in BOD_5_ in Lake Peipsi *s.s.*, while no trend was found in Secchi and PhytBM (Table 4). In Lake Pihkva, BOD_5_ and Secchi showed an increase in years 2001–2019. No significant trends were observed for Lake Lämmijärv. In six-month data, COD_5_ showed a consistent decrease in Peipsi *s.s.* over the years 2001–2019 (Fig. 4D), which was perhaps associated with changes in catchment processes. When six-month data were averaged, no trend was observed in TP in Lake Peipsi *s.s.* (Fig. 4A, Table 4), and a significant positive trend was observed in BOD_5_ in Lake Lämmijärv (Fig. 4B, Table 4), similar to what was suggested by the analysis of the Estonian monitoring data (Table 2). No changes in trend were observed for Secchi that was also suggested as a variable sensitive to sampling frequency (Fig. 4C). Long-term changes in joint Estonian-Russian monthly data in case of TP, BOD_5_ and Secchi depth in Lake Peipsi *s.s.* and Lake Lämmijärv were almost the same, as described above for Estonian data with the only exception of TP that no longer showed a decreasing trend in August in Lake Peipsi *s.s.* and Lake Lämmijärv (ESM Fig. S3). Totally different patterns in TP, BOD_5_ and Secchi were observed in Lake Pihkva. TP showed a slight decline in all months from May to September, but a notable increase in October over the years 2001–2019 (ESM Fig. S3). Monthly changes in Secchi were opposite to those in TP and resulted in overall increasing trend for the whole ice-free period (in both three and six-month data). BOD_5_ showed an increasing trend in three of the six months, including May, July and August in Lake Pihkva.

**Fig. 5:**
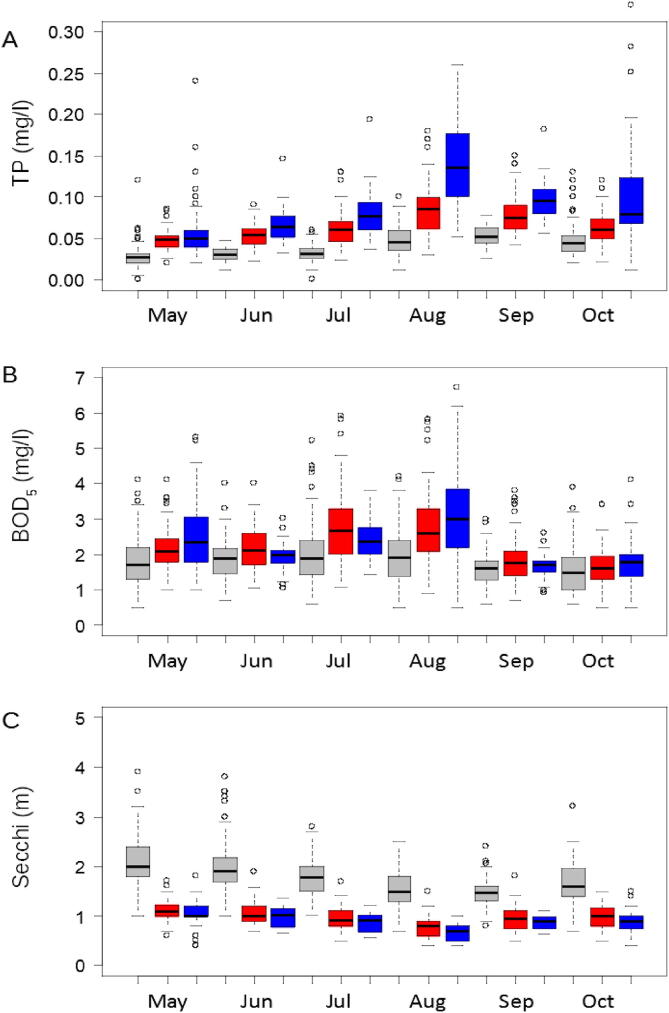
Seasonal distribution of total phosphorus (TP; A), biological oxygen demand (BOD_5_; B) and Secchi depth (Secchi; C) in Lake Peipsi *s.s.* (grey), Lake Lämmijärv (red) and Lake Pihkva (blue) for years 2001–2019. (For interpretation of the references to colour in this figure legend, the reader is referred to the web version of this article.)

Long-term averages were similar for three-month and six-month joint data. Generally, the southern basins showed more pronounced seasonality of water quality variables than Lake Peipsi *s.s.* TP increased towards summer peaking in August (Lake Lämmijärv, Lake Pihkva) and September (Lake Peipsi *s.s.*, Fig. 5A). BOD_5_ values were considerably lower in September-October than in preceding months (Fig. 5B). Seasonal changes in Secchi were opposite to those in TP (Fig. 5C). The TP, BOD_5_, COD_Cr_ and PhytBM were significantly higher, and Secchi lower in Lake Lämmijärv and Lake Pihkva than in Lake Peipsi *s.s.* (*p <* 0.001). COD_Cr_ was significantly higher in Lake Pihkva than in Lake Lämmijärv, while other studied variables were similar in the two southern basins (*p >* 0.05). Significant correlations were found between studied water quality variables (Table 5). For example, TP correlated positively with BOD_5_, COD_Cr_, PhytBM, and negatively with Secchi transparency, with increasing of the Spearman’s correlation coefficient from BOD_5_ to Secchi.

**Table 5:** Spearman’s correlations between the studied variables in a three-lake-system based on combined three- and six-months data of Estonian and Russian monitoring at a significance level *p*< 0.001. The water quality variables included biological oxygen demand (BOD_5_), dichromatic chemical oxygen demand (COD_Cr_), total phosphorus (TP), Secchi depth (Secchi), phytoplankton biomass (PhytBM).

Variable	Number of months	BOD_5_	COD_Cr_	TP	Secchi
BOD_5_					
COD_Cr_	3	0.312 (*n =* 959)			
	6	0.233 (*n =* 1900)			
TP	3	0.190 (*n =* 965)	0.417 (*n =* 959)		
	6	0.152 (*n =* 1906)	0.420 (*n =* 1900)		
Secchi	3	−0.270 (*n =* 944)	−0.503 (*n =* 936)	−0.732 (*n =* 929)	
	6	−0.250 (*n =* 1885)	−0.494 (*n =* 1877)	−0.767 (*n =* 1870)	
PhytBM	3	0.222 (*n =* 838)	0.237 (*n =* 824)	0.496 (*n =* 818)	−0.465 (*n =* 809)
	6	0.269 (*n =* 1779)	0.269 (*n =* 1765)	0.472 (*n =* 1759)	−0.464 (*n =* 1750)

## Discussion

### Consequences of reduced monitoring frequency

As a result of efforts in Estonian and Russian methods harmonisation, the methodological differences in monitoring data are likely of marginal importance. However, the sampling frequency seems to have considerable implications on water quality assessment. For example, the tendency of TP to show a long-term decreasing trend based on three-month data was not confirmed when data for June, July and September were added. A similar pattern suggested first by the Estonian data analysis appeared also in the joint data*.* TP showed a decreasing trend in two (August, October) of the three months in a three-month-data in Lake Peipsi *s.s.* that determined the overall trend in years 2001–2019. As in other shallow lakes (e.g., Søndergaard et al., 2003, Nürnberg, 2009), increases in TP approaching autumn are explained by P release from sediments in Lake Peipsi (internal P load; Tammeorg et al., 2014). Earlier studies demonstrated that internal P load in Lake Peipsi correlates positively with water temperature in August and water level in months from August to October (Tammeorg et al., 2020). Hence, a decrease in water temperature in August could reduce sediment P release through a decrease in mineralization of organic matter or reduced anoxia of the sediment surfaces (e.g., Søndergaard and Jeppesen, 2020) and cause a slight decline in TP. On the other hand, the trend was counteracted partially by a decrease in water level in August and September. This finding agrees with the previous conclusion by Tammeorg et al. (2020), who found no clear trend in long-term internal P load in Lake Peipsi (Lake Peipsi *s.s.* and Lake Lämmijärv). Therefore, a declining trend in TP was manifested in a similar trend when three-month-data were averaged, but was weakened by adding months with no clear trends in TP. By contrast, adding June, July and September data strengthened an increasing trend in BOD_5_ over the years 2001–2019 in Lake Lämmijärv, as both June and September were the months with a clear increase in BOD_5_. In general, an increase in BOD_5_ in June, September and October in long-term agrees with an increase in water temperature that enhances the production of organic material. Hence, disregarding some months of the ice-free season may lead to under- or overestimation of certain factors, and there is no consistency in response of different basins. That is why, data of the whole ice-free season are recommended for the adequate water quality assessment.

Satellite imagery has assumed a greater role in filling seasonal data gaps (Watkins, 2009, Kratzer et al., 2019, Coffer et al., 2020). More classical approaches include mathematical modelling of different complexity from simple linear to multilevel models. A number of 3D models has been elaborated for studies of seasonal and spatial dynamics of the key physical, chemical and biological processes in large lakes worldwide (Leon et al., 2012, Bocaniov et al., 2016, Soulignac et al., 2018, Zou et al., 2020). Lake Peipsi is not an exception. The most recent and comprehensive 3D model for Lake Peipsi, i.e. the heat and mass transfer model, has described, for instance, the distribution of the TP concentration in Lake Pihkva in July as a function of three major processes including circulation of water mass from Peipsi *s.s.*, “diluting” effect of the inflowing Velikaya River, and mass exchange at the water–sediment interface (Kondratyev et al., 2021). Generally, colder and denser water of Lake Peipsi *s.s.* blocks water exchange with Lake Pihkva from the north, while, depending on the winds, the P-rich water from Pihkva may penetrate to the eastern and central regions of Lake Peipsi *s.s.* (Kondratyev et al., 2021). The results of the current study for Lake Pihkva supported these patterns. It seems that diluting effect of the Velikaja River is most pronounced in the period from May to September. Moreover, it has advanced since 2001 to 2019 (decrease in TP and increase in Secchi). The P transport from Lake Pihkva affects mainly Lake Lämmijärv, but also the northernmost basin, Lake Peipsi *s.s.*, as indicated by disappearance of significant decrease in TP in August in Lämmijärv and in Lake Peipsi *s.s.* (revealed by Estonian data), when Russian data (including missing months data) were added. These specifics of Lake Pihkva are largely explained by a relatively short water residence time (0.21 y vs 1.75 y in Lake Peipsi *s.s.*). Lake Lämmijärv with a water residence time of 0.05 y is rather a flow through system. A decrease in TP over the years 2001–2019 in months from May to September may reflect a decrease in external P load from the Velikaja River associated with reduced point-source pollution. However, an increase in TP in October that appears to be crucial for the whole ice-free period (as abates the positive trend in Lake Pihkva) is likely related with an increase of P loading from diffuse sources. The losses of nutrients from fields in autumn were likely favoured by dry and hot summers (as indicated by an increase in water temperature and decrease in water level) that was a tendency of the long term studies here. Thus, omitting the same months in averaging over the ice-free season in the southern basins where nutrient dynamics are dominated by external processes may be manifested in totally different trends and influence water quality assessment.

A similarity in trends of the recent 3D heat and mass transfer model and trends observed in the current study support the reliability of monthly mean estimates. Indeed, our approach is more suitable for practical uses (e.g., in water quality assessment) due to its simplicity, as demonstrated also in other studies relying on linear models (e.g., Nürnberg, 1996, Phillips et al., 2008). We presented here a simple tool that enables to complete the picture on the spatio-temporal variability of key water quality variables, provided that there is a potential link between the months with the most limited data and most available data. This approach enables the most comprehensive use of the long-term data series for the whole Lake Peipsi in water quality assessment and management.

### Factors explaining variations in lake water quality

In general, the joint Estonian and Russian monitoring data for the ice-free period 2001–2019 did not reveal an improvement in the ecological state of the lake complex, which has been repeatedly concluded by the analyses of Estonian data, exclusively, for Lake Peipsi *s.s.* and Lake Lämmijärv (Tammeorg et al., 2016, Nõges et al., 2020, Nõges, 2020). An increase in BOD_5_ in all three basins of Lake Peipsi indicate further deterioration of lake water quality. It is the continuously high phosphorus that controls the functioning of the entire ecosystem. The long-term monitoring data of the Estonian side have demonstrated the resilience of the lake to nitrogen input, that is reflected in greater stability of TN than TP concentrations (Kangur and Möls, 2007, Buhvestova et al., 2011). The excess of nitrogen is removed from the system through denitrification. But at the time of N-limitation, during the late summer-early autumn, it is fixed from the atmosphere by cyanobacteria that dominate in phytoplankton (Nõges, 2020). Thus, it is most important to reduce further the external P loading that is still too high. The nutrient load from point sources has been reduced considerably, while the control of the nutrient input from the diffuse sources is the primary target (Nõges et al., 2007; 2020). For Lake Pihkva, the contribution of point-source pollution is also high (Zadoskaya et al., 2017).

On the other hand, rapid re-oligotrophication cannot be expected due to a large pool of legacy P in sediments, similar to what was reported for many other lakes worldwide (Jeppesen et al., 2005; McCrackin et al., 2017). Tammeorg et al. (2016) showed an increase in internal P load during the period of lower external P load (2001–2005) coinciding with the general deterioration in the lake water quality in August compared with the period of maximum human impact (1985–1989). Moreover, the internal P loading calculated based on *in situ* TP increase in the water column over the growing period is three times higher than the external TP load and still does not show a tendency towards decline (Tammeorg et al., 2020). The calculations based on the sediment profiles by Tammeorg et al. (2016) showed higher internal P load in Lake Pihkva with a higher trophic state than in Lake Peipsi *s.s.* (Tammeorg et al., 2016) that is in agreement with the numerous studies reporting that sediment P release increases with the trophic state (e.g., Nürnberg, 2009). Nevertheless, the P budget of Lake Peipsi *s.s.* is more influenced by internal P loads due to much longer water residence time.

Sediment P becomes available at the most critical time, when the external loads are low (Tammeorg et al., 2014). Phosphorus balance calculations in Lake Peipsi *s.s.* and Lake Lämmijärv demonstrated that the internal P loading depends mainly on P sedimentation (measured using sedimentation traps; Tammeorg et al., 2015). Resuspension of P accounted for the bulk of P sedimentation, i.e. resuspension is the main mechanism behind internal P load (Tammeorg et al., 2015). Also the dynamic ratio (i.e., square root of the lake surface area in square kilometres divided by its mean depth in meters; Håkanson, 1982) for the all three basins of Lake Peipsi (6.2, 6.1, and 7.0 for Lake Peipsi *s.s.*, Lake Lämmijärv and Lake Pihkva, respectively) suggest that 100% of the lake bottom is affected by sediment resuspension. Moreover, larger implications are expected in Lake Pihkva than in Lake Peipsi *s.s.* based on the dynamic ratio values comparison. Low water levels promote sediment resuspension (Tammeorg et al., 2013). Resuspension brings large amounts of particulate P back to the water column. This P can become bioavailable as soon as it reaches the surface water layers with high pH values where hydroxyl groups displace phosphates by attaching them to iron or aluminium in sediment particles in the ligand-exchange reactions (Koski-Vähälä and Hartikainen, 2001).

Moreover, resuspension in Lake Peipsi was shown to cause significant disturbances in the surface layers of bottom sediments, and lead to an increase in the concentration gradient, and the subsequent release of P from deeper P-rich layers (Tammeorg et al., 2016, Tammeorg et al., 2020). Under anaerobic conditions in deeper sediment layers, ferric iron is reduced to ferrous iron and phosphates are released into the pore water, as the classical model of P release describes (Einsele, 1936, Mortimer, 1941, Mortimer, 1942). Further, resuspension promotes the transport of P to the water column via interaction with the redox-related release. In zones of organic matter accumulation, mineralisation also contributes to the internal P load. Despite oxygen deficiency in the bottom water layer was rarely observed in Lake Peipsi during the monitoring, our data indicate a high potential for P release from anoxic sediment surfaces (Tammeorg et al., 2020). The likelihood of this phenomenon increases with increasing water temperature that promotes settling of organic matter and increased rates of oxygen consumption by microbial respiration (Wilhem, 2014). Thus, sediment resuspension and organic matter mineralisation pump out P from sediments.

The lack of trends in water quality variables in the current study may be explained also by considerable fluctuations in weather conditions. In the current study, we showed that decrease in water temperature in August in long-term may not favour sediment P release, but the tendency is reversed by a decrease in water level that supports sediment P release. Similarly, weather conditions were often reported to amplify the eutrophication symptoms (e.g., Jeppesen et al., 2009, Spears et al., 2021). In Lake Peipsi, a role of exceptional weather conditions, i.e. low water level and high water temperature for whole lake ecosystem is well-documented (Kangur et al., 2005, Kangur et al., 2013). Nõges (2020) demonstrated that the P content must be 35% less at low water levels than at high water levels in order to achieve an improvement indicated by phytoplankton.

This is the first study that presents the results of shared monitoring data for Lake Peipsi. Indeed, this is one of the most complicated cases (among the examples presented by e.g., Servos et al. (2013)), as there are large contextual differences in the sharing countries, including jurisdiction, water quality monitoring and assessment approaches. Huge data harmonisation efforts led by the Joint Comission enabled data sharing and combination. The analysis of the data presented here provides an important basis for shared water quality assessment. Furthermore, the analysis facilitated discussion of the factors affecting water quality at the scale of the whole lake, i.e. at a higher level than in previous studies. Both of these aspects are highly important for the management of lake water quality that can be achieved only through transboundary cooperation. Hence, data sharing and shared monitoring are vital for management of transboundary lakes.

## Conclusions

The consistency of Estonian and Russian monitoring data facilitates their joint analysis for the purposes of ecological state assessment of the entire large transboundary Lake Peipsi. Estonian data analysis suggested that water quality assessment results (e.g. long-term trends) may be sensitive to sampling frequency in case of total phosphorus (TP), biological oxygen demand (BOD_5_), and Secchi depth. Disregarding some months of the ice-free season may lead to under- or overestimation of certain factors and there is no consistency in response of different basins. That is why, data of the entire ice-free season, and if resources permit, year-round are recommended for the adequate water quality assessment. Furthermore, we demonstrated that values of the water quality variables in frequently sampled months (May, August, October) are linked to those in months with the most limited data (June, July, September). We used this linkage to compensate for the gaps in the long-term monitoring and compiled a dataset for the whole Lake Peipsi enabling most comprehensive use of it in water quality assessment and management. In general, long-term data did not reveal any improvement of the ecological state of Lake Peipsi. In the light of the continuously high phosphorus load, which effects will be amplified by climate change, reduction of the external nutrient load gains more importance for improving the ecological status of the lake.

## Declaration of Competing Interest

The authors declare that they have no known competing financial interests or personal relationships that could have appeared to influence the work reported in this paper.

## References

[b0005] Bartram J., Ballance R. (1996). Water quality monitoring: a practical guide to the design and implementation of freshwater quality studies and monitoring programmes.

[b0010] Blank K., Haberman J., Haldna M., Laugaste R. (2009). Effect of winter conditions on spring nutrient concentrations and plankton in a large shallow Lake Peipsi (Estonia/Russia). Aquat. Ecol..

[b0015] Bocaniov S.A., Leon L.F., Rao Y.R., Schwab D.J., Scavia D. (2016). Simulating the effect of nutrient reduction on hypoxia in a large lake (Lake Erie, USA-Canada) with a three-dimensional lake model. J. Great Lakes Res..

[b0020] Buhvestova O., Kangur K., Haldna M., Möls T. (2011). Nitrogen and phosphorus in Estonian rivers discharging into Lake Peipsi: estimation of loads and seasonal and spatial distribution of concentrations. Est. J. Ecol..

[b0025] Coffer M.M., Schaeffer B.A., Darling J.A., Urquhart E.A., Salls W.B. (2020). Quantifying national and regional cyanobacterial occurrence in US lakes using satellite remote sensing. Ecol. Ind..

[b0030] Einsele W. (1936). Über die Beziehungen des Eisenkreislaufs zum Phosphatkreislauf im eutrophen See. Arch. für Hydrobiol..

[bib227] Håkanson L (1982). Lake bottom dynamics and morphometry: the dynamic ratio. Water Resources Research.

[b0035] Herdendorf C.E. (1982). Large lakes of the world. J. Great Lakes Res..

[b0040] Iital A., Stålnacke P., Deelstra J., Loigu E., Pihlak M. (2005). Effects of large-scale changes in emissions on nutrient concentrations in Estonian rivers in the Lake Peipsi drainage basin. J. Hydrol..

[b0045] Jeppesen E., Kronvang B., Meerhoff M., Søndergaard M., Hansen K.M., Andersen H.E. (2009). Climate change effects on runoff, catchment phosphorus loading and lake ecological state, and potential adaptations. J. Environ. Qual..

[b0050] Kangur K., Kangur A., Kangur P., Laugaste R. (2005). Fish kill in Lake Peipsi in summer 2002 as a synergistic effect of a cyanobacterial bloom, high temperature, and low water level. Est. J. Ecol..

[b0055] Kangur K., Kangur P., Ginter K., Orru K., Haldna M., Möls T., Kangur A. (2013). Long-term effects of extreme weather events and eutrophication on the fish community of shallow Lake Peipsi (Estonia/Russia). J. Limnol..

[b0060] Kangur K., Möls T. (2007). European Large Lakes Ecosystem changes and their ecological and socioeconomic impacts.

[b0065] Kondratyev S.A., Golosov S.D., Shmakova M.V., Ershova A.A., Zverev I.S., Ivanova E.V., Korobchenkova K.D. (2021). System of models for assessment and forecast of heat-and mass-transfer in the system “catchment-watercourse-water body”. IOP Conf. Series: Materials Sci. Engineer.

[b0070] Koski-Vähälä J., Hartikainen H. (2001). Assessment of the risk of phosphorus loading due to resuspended sediment. J. Environ. Qual..

[b0075] Kratzer S., Kyryliuk D., Edman M., Philipson P., Lyon S.W. (2019). Synergy of Satellite, In Situ and Modelled Data for Addressing the Scarcity of Water Quality Information for Eutrophication Assessment and Monitoring of Swedish Coastal Waters. Remote Sens..

[b0080] Leon L.F., Smith R.E., Malkin S.Y., Depew D., Hipsey M.R., Antenucci J.P. (2012). Nested 3D modeling of the spatial dynamics of nutrients and phytoplankton in a Lake Ontario nearshore zone. J. Great Lakes Res..

[b0085] Loigu E., Leisk Ü., Iital A., Pachel K., Haberman J., Timm T., Raukas A. (2008). Peipsi.

[bib226] McCrackin M.L., Jones H.P., Jones P.C., Moreno-Mateos D. (2017). Recovery of lakes and coastal marine ecosystems from eutrophication: A global meta‐analysis. Limnology and Oceanography.

[b0090] Mortimer C.H. (1941). The exchange of dissolved substances between mud and water in lakes. J. Ecol..

[b0095] Mortimer C.H. (1942). The exchange of dissolved substances between mud and water in lakes. J. Ecol..

[b0100] Nõges, P., 2020. Uuring Peipsi järve füüsikalis-keemiliste ja fütoplanktoni kvaliteedi-näitajate klassipiiride täpsustamiseks.

[b0105] Nõges T., Janatian N., Laugaste R., Nõges P. (2020). Post-soviet changes in nitrogen and phosphorus stoichiometry in two large non-stratified lakes and the impact on phytoplankton. Global Ecol. Conserv..

[b0110] Nürnberg G.K. (1996). Trophic state of clear and colored, soft-and hardwater lakes with special consideration of nutrients, anoxia, phytoplankton and fish. Lake Reserv. Manage..

[b0115] Nürnberg G.K. (2009). Assessing internal phosphorus load–problems to be solved. Lake Reserv. Manage..

[b0120] Ozersky T., Bramburger A.J., Elgin A.K., Vanderploeg H.A., Wang J., Austin J.A. (2021). The changing face of winter: Lessons and questions from the Laurentian Great Lakes. Journal of Geophys. Res. Biogeosci..

[b0125] Phillips G., Pietiläinen O.P., Carvalho L., Solimini A., Lyche Solheim A., Cardoso A.C. (2008). Chlorophyll–nutrient relationships of different lake types using a large European dataset. Aquatic Ecol..

[b0130] Schindler D.W., Carpenter S.R., Chapra S.C., Hecky R.E., Orihel D.M. (2016). Reducing phosphorus to curb lake eutrophication is a success. Environ. Sci. Technol..

[b0135] Servos M.R., Munkittrick K.R., Constantin G., Mngodo R., Aladin N., Choowaew S. (2013). Science and management of transboundary lakes: Lessons learned from the global environment facility program. Environ. Development.

[b0140] Sharma S., Blagrave K., Magnuson J.J., O’Reilly C.M., Oliver S., Batt R.D. (2019). Widespread loss of lake ice around the Northern Hemisphere in a warming world. Nat. Climate Change.

[b0145] Søndergaard M., Jensen J.P., Jeppesen E. (2003). Role of sediment and internal loading of phosphorus in shallow lakes. Hydrobiol..

[b0150] Søndergaard M., Jeppesen E., Steinman A.D., Spears B.M. (2020). Internal phosphorus loading in lakes: causes, case studies, and management.

[b0155] Soulignac F., Danis P.A., Bouffard D., Chanudet V., Dambrine E., Guénand Y. (2018). Using 3D modeling and remote sensing capabilities for a better understanding of spatio-temporal heterogeneities of phytoplankton abundance in large lakes. J. Great Lakes Res..

[b0160] Spears B.M., Chapman D., Carvalho L., Rankinen K., Stefanidis K., Ives S. (2021). Assessing multiple stressor effects to inform climate change management responses in three European catchments. Inland Waters.

[b0165] Spears B.M., Futter M.N., Jeppesen E., Huser B.J., Ives S., Davidson T.A. (2017). Ecological resilience in lakes and the conjunction fallacy. Nat. Ecol. Evolut..

[b0170] Tammeorg O., Horppila J., Laugaste R., Haldna M., Niemistö J. (2015). Importance of diffusion and resuspension for phosphorus cycling during the growing season in large, shallow Lake Peipsi. Hydrobiol..

[b0175] Tammeorg O., Horppila J., Tammeorg P., Haldna M., Niemistö J. (2016). Internal phosphorus loading across a cascade of three eutrophic basins: a synthesis of short-and long-term studies. Sci. Total Environ..

[b0180] Tammeorg O., Möls T., Kangur K. (2014). Weather conditions influencing phosphorus concentration in the growing period in the large shallow Lake Peipsi (Estonia/Russia). J. Limnol..

[b0185] Tammeorg O., Möls T., Niemistö J., Holmroos H., Horppila J. (2017). The actual role of oxygen deficit in the linkage of the water quality and benthic phosphorus release: potential implications for lake restoration. Sci. Total Environ..

[b0190] Tammeorg O., Niemistö J., Möls T., Laugaste R., Panksep K., Kangur K. (2013). Wind-induced sediment resuspension as a potential factor sustaining eutrophication in large and shallow Lake Peipsi. Aquat. Sci..

[b0195] Tammeorg O., Nürnberg G., Horppila J., Haldna M., Niemistö J. (2020). Redox-related release of phosphorus from sediments in large and shallow Lake Peipsi: Evidence from sediment studies and long-term monitoring data. J. Great Lake. Res..

[b0200] Utermöhl H. (1958). Zur Vervollkommnung der quantitativen Phytoplankton Methodik. Internat. Verein. fuer Limnol..

[b0205] Watkins J.M. (2009). Comparison of shipboard and satellite measurements of surface water temperature and chlorophyll a in Lake Ontario. Aquat. Ecosyst. Health Manage..

[b0210] Weyhenmeyer G.A. (2001). Warmer winters: are planktonic algal populations in Sweden's largest lakes affected?. Ambio.

[b0215] Wilhelm S.W., LeCleir G.R., Bullerjahn G.S., McKay R.M., Saxton M.A., Twiss M.R., Bourbonniere R.A. (2014). Seasonal changes in microbial community structure and activity imply winter production is linked to summer hypoxia in a large lake. FEMS Microbiol. Ecol..

[b0220] Zadoskaya O., Vuglinsky V., Gurevich E., Alekseev L., Skakalsky B., Eremeeva A., Dubrovskaya K. (2017). External and internal nutrient loads to Lake Peipsi (Chudsko-Pskovskoe) lake and ways of their reduction. Report of the analysis of the contemporary trophic state of Lake Peipsi. State Hydrol Institute.

[b0225] Zou R., Wu Z., Zhao L., Elser J.J., Yu Y., Chen Y., Liu Y. (2020). Seasonal algal blooms support sediment release of phosphorus via positive feedback in a eutrophic lake: Insights from a nutrient flux tracking modeling. Ecol. Model..

