# Supplementary material for: Opportunities for combining data of Estonian and Russian monitoring to reflect on water quality in large transboundary Lake Peipsi
Source: J Great Lakes Res. 2022 Aug;48(4):961–70. doi: 10.1016/j.jglr.2022.05.009 (PMC9353880; doi:10.1016/j.jglr.2022.05.009)
Supplement: Supplementary data 1 [file mmc1.docx]

Electronic Supplementary Material

**Opportunities for combining data of Estonian and Russian monitoring to reflect on water quality in large transboundary Lake Peipsi**

Olga Tammeorg,^a,b*^, Lea Tuvikene^a^, Sergey Kondratyev^c^, Sergey Golosov^c^, Ilya Zverev^c^, Olga Zadonskaya^d^, Peeter Nõges^a^

^a^Chair of Hydrobiology and Fishery, Estonian University of Life Sciences, Kreutzwaldi 5, 51006 Tartu, Estonia

^b^Ecosystems and Environment Research Programme, Faculty of Biological and Environmental Sciences, University of Helsinki, Viikinkaari 1, 00014 Helsinki, Finland

^c^Institute of Limnology, Russian Academy of Sciences, ul. Sevast’yanova 9, St. Petersburg, 199105, Russia

^d^State Hydrological Institute, 23, 2-ia liniia V.O., St. Petersburg, 199053, Russian Federation

*Corresponding author e-mail address: [olga.tammeorg@helsinki.fi](mailto:olga.tammeorg@helsinki.fi); +37255620835


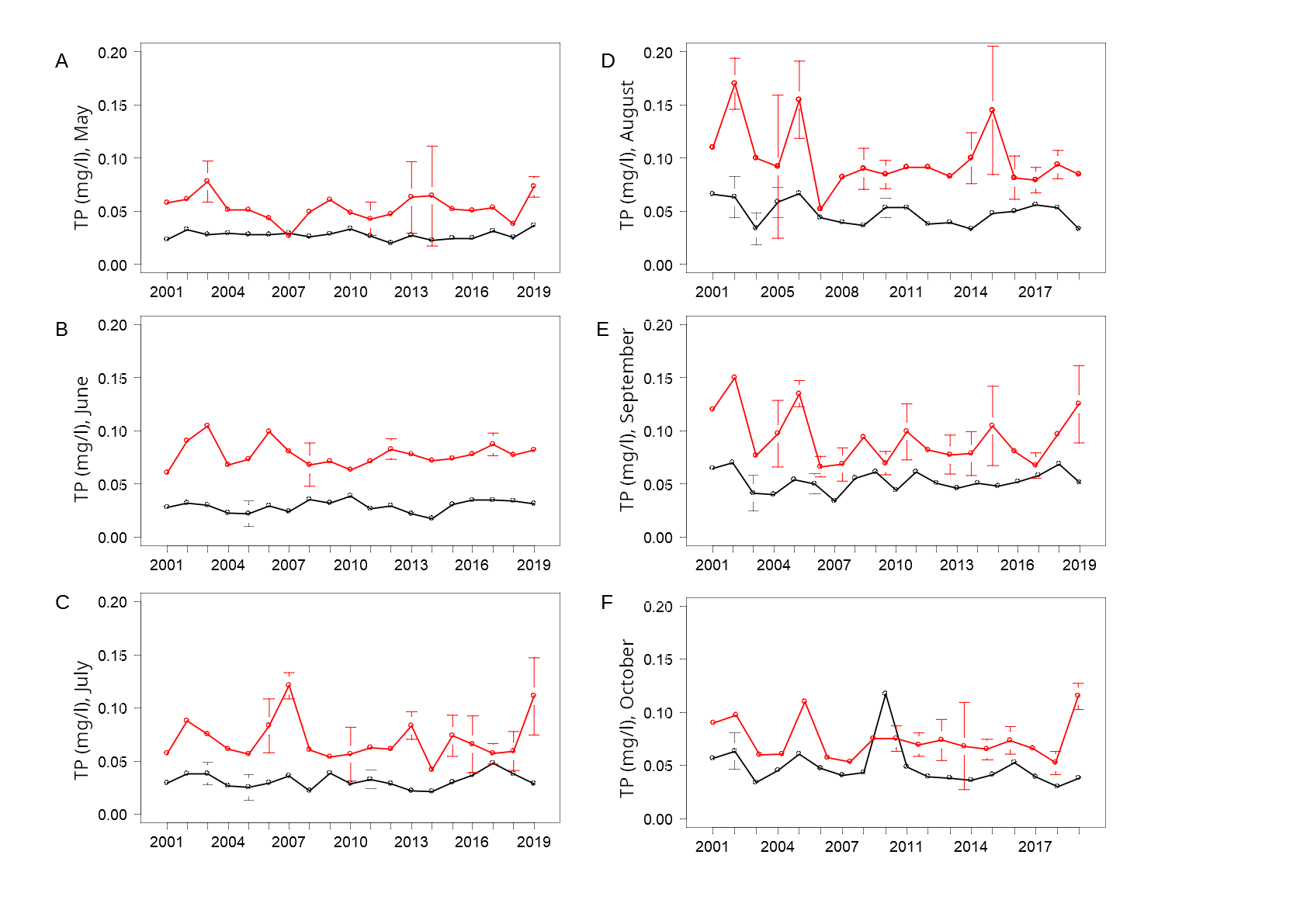


**Fig. S1.** Changes in TP concentration (mean ± 75% confidence limits) in months from May to October (A-F) in Lake Peipsi *s.s.* and Lake Lämmijärv over the years 2001-2019 based on Estonian monitoring data.


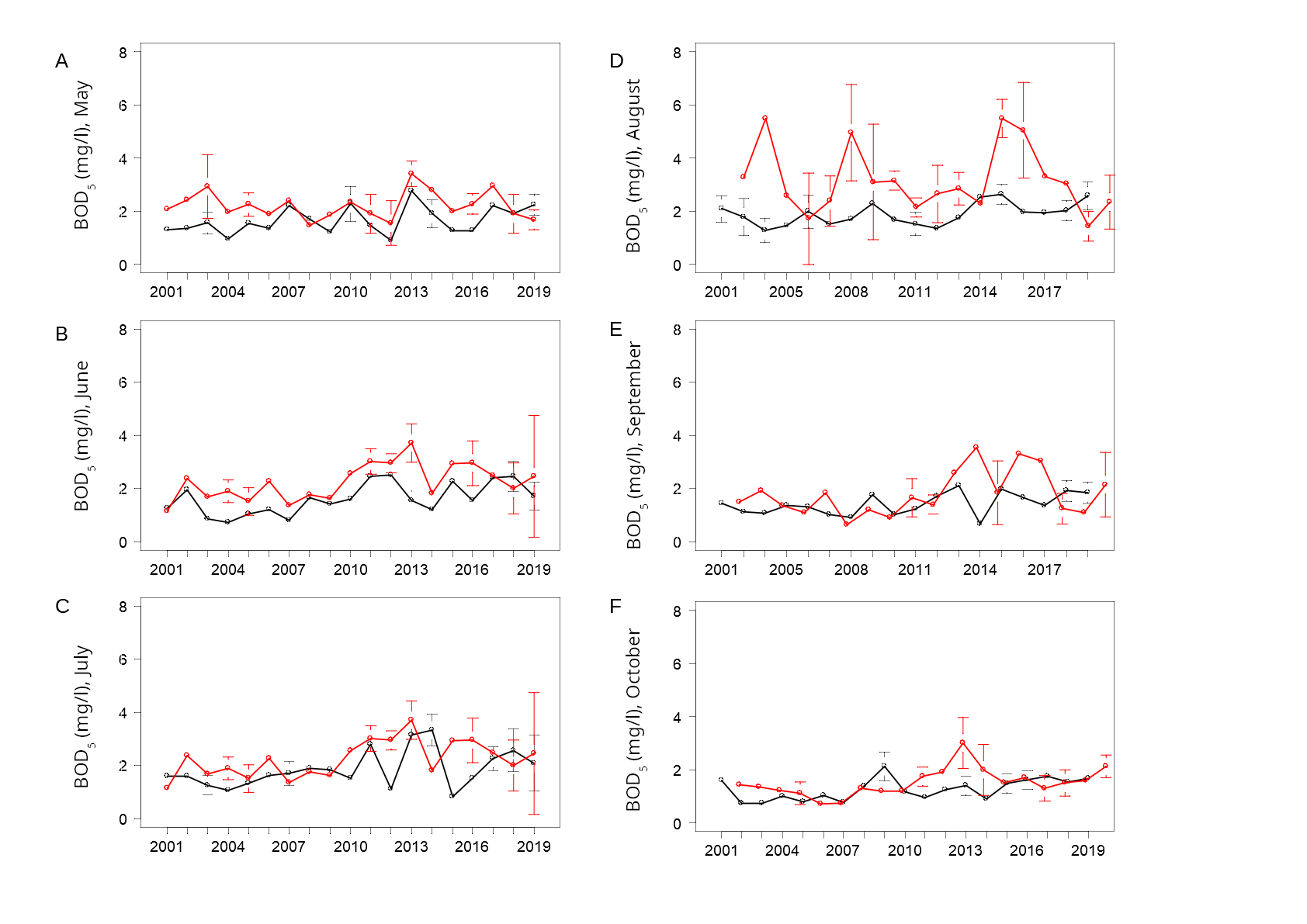

**Fig. S2.** Changes in BOD_5_ (mean ± 75% confidence limits) in months from May to October (A-F) in Lake Peipsi *s.s.* and Lake Lämmijärv over the years 2001-2019 based on Estonian monitoring data.


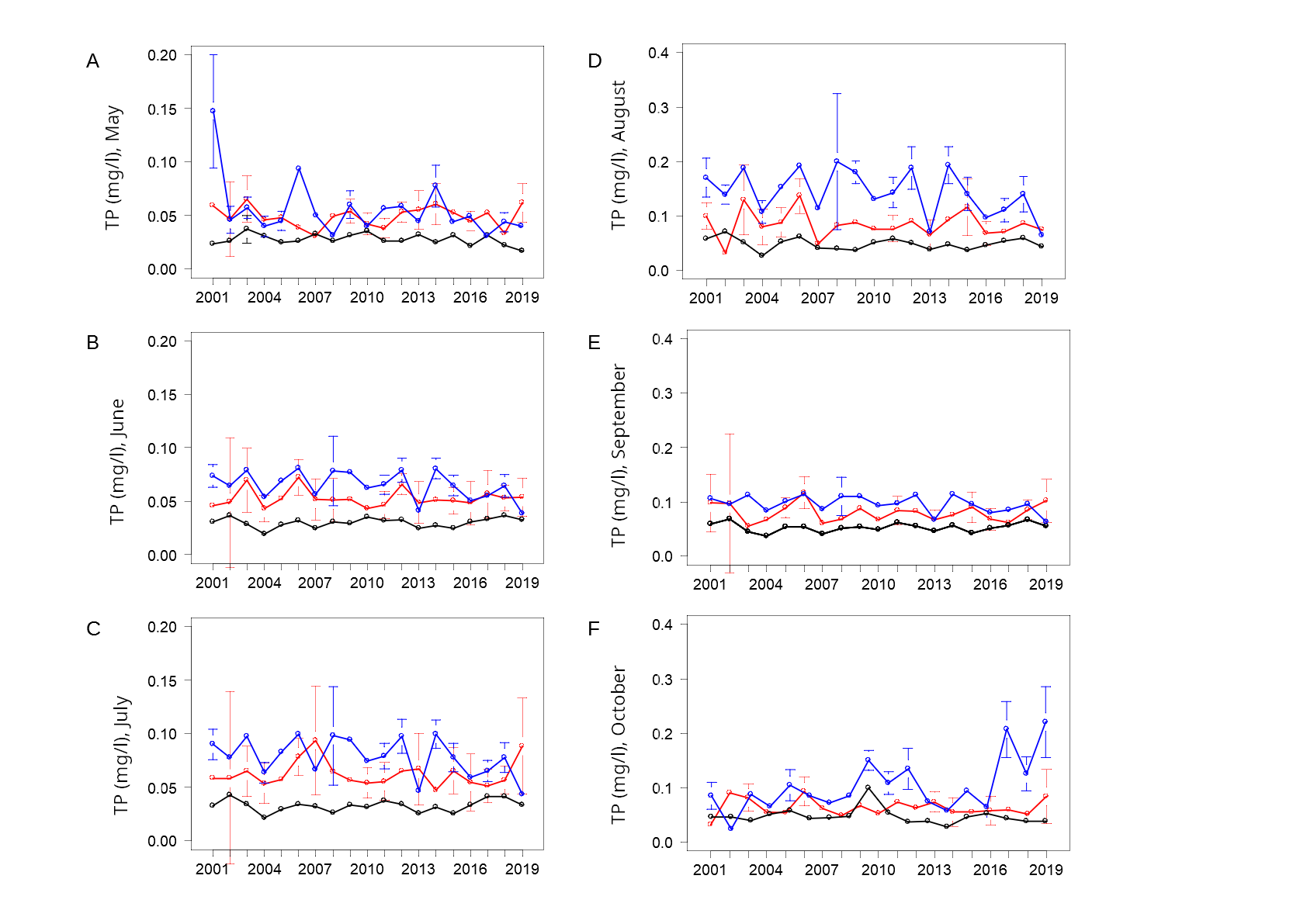


**Fig. S3.** Changes in TP concentration (mean 75% confidence limits) in months from May to October (A-F) in Lake Peipsi *s.s.*, Lake Lämmijärv and Lake Pihkva over the years 2001-2019 based on Estonian-Russian joint monitoring data. Values for the Russian monitoring sites for June, July and September were predicted.

**Table S1.** Trends (positive, negative and not significant) in water level and water temperature in May to October over the years 2001-2019. Significance level is indicated with asterisk (*p <* 0.05^*^, *p <* 0.01^**^, *p <* 0.001^***^).

|  | Water temperature (°C) | | Water level (cm) | | |
| --- | --- | --- | --- | --- | --- |
| Month | Mean (SD) | Trend (°C/y) | Mean (SD) | Trend (cm/y) | *n* |
| May | 12.58 (3.33) | +0.11^***^ | 220 (38) | -0.77^**^ | 587 |
| June | 17.15 (2.37) | +0.04^*^ | 208 (32) | -1.22^***^ | 568 |
| July | 20.66 (2.91) | -0.15^***^ | 194 (30) | -1.50^***^ | 587 |
| August | 19.32 (2.65) | -0.06^**^ | 178 (27) | -1.12^***^ | 587 |
| September | 13.73 (2.55) | +0.11^***^ | 168 (29) | -0.43^*^ | 568 |
| October | 6.86 (3.09) | +0.08^***^ | 160 (31) | n.s. | 586 |
